# Contrasting *Plasmodium* infection rates and insecticide susceptibility profiles between the sympatric sibling species *Anopheles parensis* and *Anopheles funestus s.s*: a potential challenge for malaria vector control in Uganda

**DOI:** 10.1186/1756-3305-7-71

**Published:** 2014-02-17

**Authors:** Charles Mulamba, Helen Irving, Jacob M Riveron, Louis G Mukwaya, Josephine Birungi, Charles S Wondji

**Affiliations:** 1 Vector Biology Department, Liverpool School of Tropical Medicine, Pembroke Place, Liverpool L3 5QA, UK; 2 Uganda Virus Research Institute, Entebbe, Uganda

**Keywords:** *An. parensis*, *An. funestus*, Malaria, Insecticide resistance, Vector control, Uganda

## Abstract

**Background:**

Although the *An. funestus* group conceals one of the major malaria vectors in Africa, little is known about the dynamics of members of this group across the continent. Here, we investigated the species composition, infection rate and susceptibility to insecticides of this species group in Uganda.

**Methods:**

Indoor resting blood-fed Anopheles adult female mosquitoes were collected from 3 districts in Uganda. Mosquitoes morphologically belonging to the *An. funestus* group were identified to species by PCR. The sporozoite infection rates were determined by TaqMan and a nested PCR. Susceptibility to major insecticides was assessed using WHO bioassays. The potential role of four candidate resistance genes was assessed using qRT-PCR.

**Results:**

*An. funestus s.s*. and *An. parensis*, were the only members of the *An. funestus* group identified. Both species were sympatric in Masindi (North-West), whereas only *An. parensis* was present in Mityana (Central) and Ntungamo (South-West). The *Plasmodium falciparum* infection detected in *An. parensis* (4.2%) by TaqMan could not be confirmed by nested PCR, whereas the 5.3% infection in *An. funestus* s.s. was confirmed. *An. parensis* was susceptible to most insecticides, however, a moderate resistance was observed against deltamethrin and DDT. In the sympatric population of Masindi, resistance was observed to pyrethroids (permethrin and deltamethrin) and DDT, but all the resistant mosquitoes belonged to *An. funestus s.s*. No significant over-expression was observed for the four P450 candidate genes CYP6M7, CYP9K1, CYP6P9 and CYP6AA4 between deltamethrin resistant and control *An. parensis*. However, when compared with the susceptible FANG *An. funestus s.s* strain, the CYP9K1 is significantly over-expressed in *An. parensis* (15-fold change; P < 0.001), suggesting it could play a role in the deltamethrin resistance.

**Conclusion:**

The contrasting infection rates and insecticide susceptibility profiles of both species highlights the importance of accurate species identification for successful vector control programs.

## Background

Malaria control in Africa, notably in Uganda, has seen a significant scale up of vector control interventions such as Long Lasting Insecticide Nets (LLINs) and Indoor Residual Spraying (IRS) [1]. The monitoring of the efficacy of these programs requires a good understanding of the vector composition in areas under control. One of the main malaria vectors in Uganda, *Anopheles funestus s.s.* has been found to be resistant to both pyrethroids and DDT in Eastern Uganda [2], highlighting the need to closely monitor this vector to ensure the continued success of control interventions. However, *An. funestus* belongs to the *Anopheles funestus* group which comprises nine to eleven morphologically indistinguishable species at the adult stage; *An. funestus s.s, An. parensis, An. confusus, An. funestus-like, An. aruni, An. vaneedeni, An. leesoni, An. brucei, An. rivulorum, An. rivulorum-like, and An. fuscivenosus*[3-7]*. An. funestus s.s, An. parensis*, *An. vaneedeni* and *An. aruni Sobti* have identical morphology at all or some life stages and are referred to as the *funestus* sub-group [4,5]. The other members of the group exhibit distinctive characteristics at different stages [8,9].

*An. funestus s.s.* and *An. rivulorum* are distributed throughout sub-Saharan Africa [4,5], *An. parensis* is common in South Africa, Swaziland and eastern Africa, whereas the other members are more localized [8,9].

*An. funestus s.s* is the most efficient malaria vector in this group [10] and one of the major vectors in Uganda [11], while the other group members are known to be zoophilic with only *An. rivulorum* having been implicated as a minor vector in Tanzania [10]. *An. vaneedeni* has been shown to transmit parasites under laboratory conditions but has not been associated with malaria transmission in nature [12].

Due to the significant differences existing between the members of the *An. funestus* group in terms of their vectorial capacities, resting and biting behaviour and despite their close morphological similarities [13], it is important to accurately identify mosquitoes of this group and establish their geographical distribution. This is particularly important in order to assess their respective contribution to malaria transmission and their susceptibility to insecticides used by control programs. In Uganda, if *An. funestus s.s.* has been previously described in the country, little is known about the existence of other members of the *An. funestus* group. Their presence, geographical distribution, their resting behaviour, their vectorial capacity and their susceptibility to main insecticides used in public health remain poorly characterised. In an attempt to fill these gaps of knowledge, we report here the characterisation of the *An. funestus* group from 3 locations in Uganda, particularly their species composition, their contribution to malaria transmission and their susceptibility to main public health insecticides.

## Methods

### Study sites

**Figure 1:**
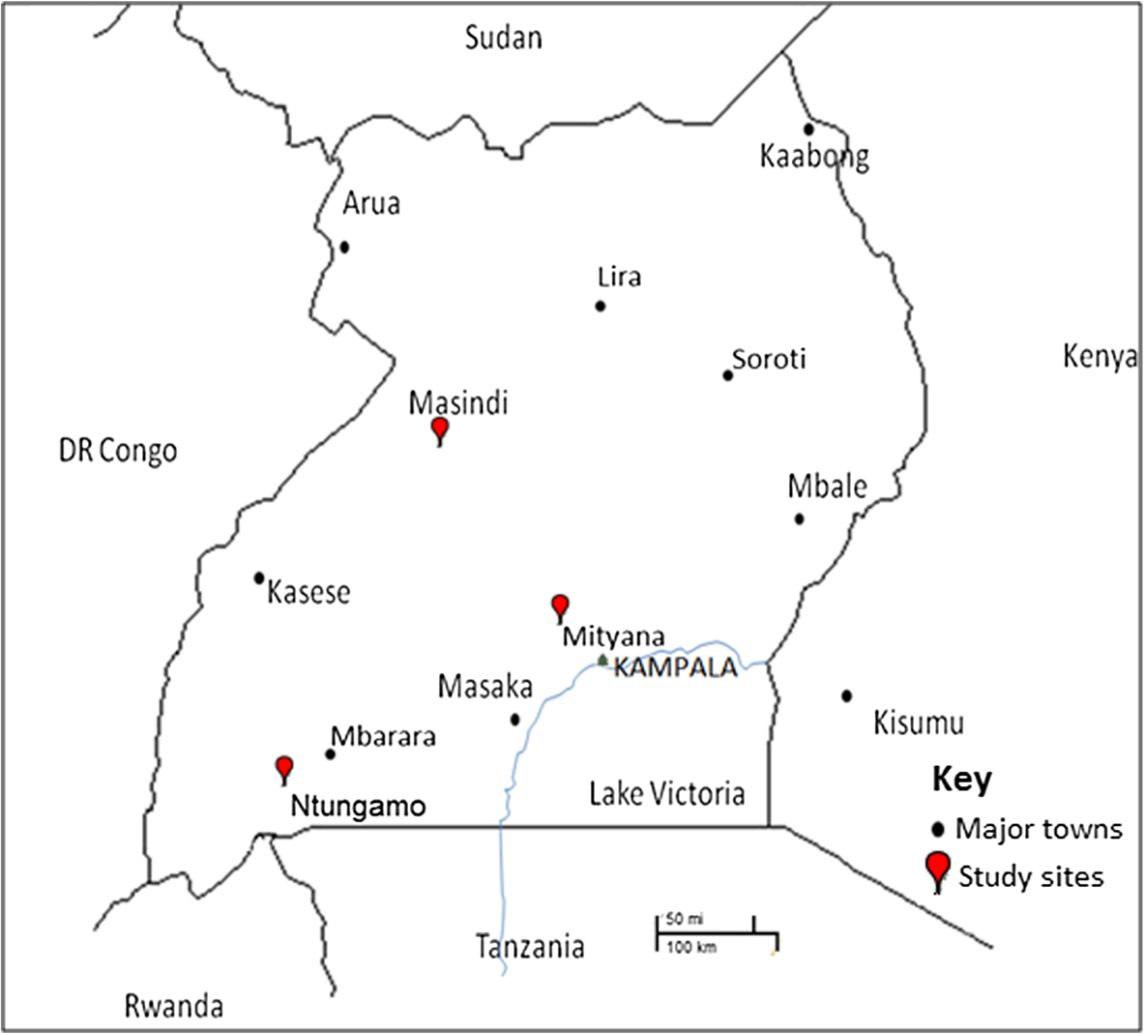
**Map of Uganda showing the three study districts (with the red indicator).** Major towns in Uganda are also included for further guidance.

Adult *Anopheles* mosquitoes were collected from 3 districts in Uganda (Figure 1); Masindi (1.68°N, 31.70°E), Mityana (0.40°N, 32.03°E) and Ntungamo (0.88°N, 30.26°E) as part of an insecticide resistance monitoring program. Villages sampled in these districts are located in close proximity with rivers, swamps and tributaries joining major rivers as these permanent water bodies are suitable breeding sites for *An. funestus*. The surrounding vegetation is mainly shrubs, maize crops, finger-millet, banana and coffee plantations. Fishing and subsistence farming are the main human activities around these villages. It was of notice that domesticated animals including cattle, goats and sheep were present but on a small scale.

### Mosquito collection and rearing

Indoor resting blood fed adult female Anopheles mosquitoes (F_0_s) were collected between 06.00 am and 12.00 pm, following verbal consent from the village Local Council 1 (L.C1) chairpersons and household owners. Mosquito collections were carried out between early May and June, 2012. Mosquitoes were collected into netted paper cups using manual aspirators and torches, kept in a cool box and immediately transported to the insectary at the Uganda Virus Research Institute based in Entebbe, Uganda.

A forced-egg laying method described by [2] was used to induce the females to lay eggs. Eggs were stored at room temperature for up to 3 days and were transferred to the Liverpool School of Tropical Medicine (LSTM), UK (under the LSTM import license from DEFRA). The egg batches were allowed to hatch in a small paper cup and later transferred to larvae bowls for rearing as previously described [2,14].

### Species Identification

Field caught females (F_0_s) which oviposited first generation (F_1_) egg batches above, were morphologically identified as belonging to *An. funestus* group according to the key of [4]. The F_0_s were split into two portions (head + thorax and abdomen). Genomic DNA was extracted from head and thorax (including proboscis) using the Livak method described in [15], while the abdomen was reserved for possible future investigations. A cocktail PCR described by [16] was performed to identify member species of the *An. funestus* group with the use of an *An. funestus* specimen as positive control.

### Estimation of the sporozoite infection rate

The sporozoite infection rate was determined using the TaqMan assay described by [17]. The real-time PCR MX 3005 (Agilent, Santa) system was used for amplification. 1 μl of gDNA for each sample was used as a template in a 3-step program, with a denaturation at 95°C for 10 mins, followed by 40 cycles of 15 sec at 95°C and 1 min at 60°C. Primers described by [17] were used together with two probes labelled with fluorophores, FAM to detect *Plasmodium falciparum*, and HEX to detect *P. ovale, P. vivax and P. malariae*. Two *P. falciparum* samples and a mix of *P. ovale, P. vivax and P. malariae* were used as positive controls.

A nested PCR was performed for all the positive samples to validate the TaqMan assay. Two amplification reactions were carried out using cycling parameters of; 95°C for 5 min, 25 cycles of: 94°C for 30 sec, 58°C for 2 min, 72°C for 2 min, final extension at 72°C for 5 min. Primers rPLU 5, rPLU 6 were used during the first amplification reaction and P.fal1, P.fal2 were used for the second amplification reaction as described by [18].

### Insecticide susceptibility assays

Following WHO procedures [19], F_1_ adults aged between 2–5 days were exposed for 1 hour to insecticide impregnated papers at WHO recommended concentrations. Eight insecticides belonging to the four major public health classes of insecticide were tested: the pyrethroids permethrin (0.75%), deltamethrin (0.05%), lambda-cyhalothrin (0.05%) and 0.05% etofenprox; the organochlorines DDT (4%) and dieldrin (4%); the carbamate bendiocarb (0.1%) and the organophosphate malathion (5%). To further assess the extent of the susceptibility levels to all the insecticides, the F_1_ population from Mityana was tested at two more exposure times of 30 and 20 minutes. Each test included control mosquitoes exposed to non treated papers.

### Transcription profiling of candidate resistance genes

A quantitative Reverse Transcriptase PCR (qRT-PCR) was performed to assess the potential role of previously detected detoxification genes in *An. funestus s.s*. in the resistance observed in the collected *An. funestus* group mosquitoes. Total RNA was extracted from three batches (R1–R3) of 10 F_1_ females (2–5 days old) that survived after 30 minutes exposure to deltamethrin from Mityana. RNA was also extracted from 3 batches (C1-C3) of unexposed F_1_ females that were used as control samples. Additionally, RNA was also extracted from 3 batches of 10 female mosquitoes from the fully susceptible *An. funestus s.s* strain FANG (S1-S3). RNA extraction, cDNA synthesis and qRT-PCR reactions were performed as previously reported [20,21]. Expression and fold change of each gene in resistant (R), control (C) and Fang susceptible (S) were calculated according to 2^-ΔΔCT^ method [22] following normalization with housekeeping genes RSP7 ribosomal protein S7 (AGAP010592) and the Actin 5C (AGAP000651) genes [20].

## Results

### Mosquito collection and rearing

A total of 1135 F_0_ mosquitoes were collected from the 3 villages combined with 97.97% belonging to the *An. funestus* group. The highest number of F_0_s came from Mityana (n = 688), whereas fewer samples were collected from Ntungamo (n = 196). 251 F_0_s were collected from Masindi. A total of 407 F_1_ egg batches were obtained for generating F_1_ adults in the insectary. F_0_s reported here were identified as belonging to the *An. funestus* group after performing morphological assessment of collected samples. Other Anopheles species mainly from the *An. gambiae* complex were also found in the study areas, notably; Masindi (27 F_0_s), Mityana (12 F_0_s) and Ntungamo (19 F_0_s). No attempts were made to obtain egg batches from non-members of the *An. funestus* group.

### Species Identification and distribution

The species ID PCR performed indicated that *An. funestus s.s*. and *An. parensis*, were the only members of the *An. funestus* group collected from the three locations. *An. parensis* was the only species found in Mityana (Mt) and Ntungamo (Nm), whereas for Masindi (Ms) both *An. funestus* and *An. parensis* were present in a ratio of almost 1:1 (Table 1).

**Table 1:** Species composition by location after PCR species ID

**Description**	**Location**		
	**Mityana**	**Ntungamo**	**Masindi**
Number of F0s Identified	200	170	97
Number of *An. funestus*	0	0	42
Number of *An. parensis*	197	162	55

3 negative PCR recorded in Mityana and 8 in Ntungamo.

### *Plasmodium* infection rates

A total of 140 samples (38 for *An. funestus* and 102 for *An. parensis*) from Mityana and Masindi were tested for sporozoite infection using TaqMan. In Mityana, all the 94 mosquitoes tested were *An. parensis* exhibiting 4 *Plasmodium falciparum* infections (4.2%), whereas in Masindi, all positive samples were *An. funestus s.s* with an infection rate of 2/38 (5.3%), (Table 2). *Plasmodium falciparum* was the only detected malaria parasite species in all locations. However, when the nested PCR [18] was carried out, none of the *An. parensis* from Mityana was positive, whereas the two *An. funestus s.s.* from Masindi remained positive.

**Table 2:** Malaria species distribution by location by TaqMan

**Location**		**Number of F0s tested**	**Number of positives**	**% positivity**	**Detected *Plasmodium *** **species**
**Masindi**	*An. funestus*	38	2	4.3	*Plasmodium falciparum*
	*An. parensis*	8	none	0.0	*_*
**Mityana**	*An. parensis*	94	4	4.3	*Plasmodium falciparum*

### Susceptibility to Insecticides

**Figure 2:**
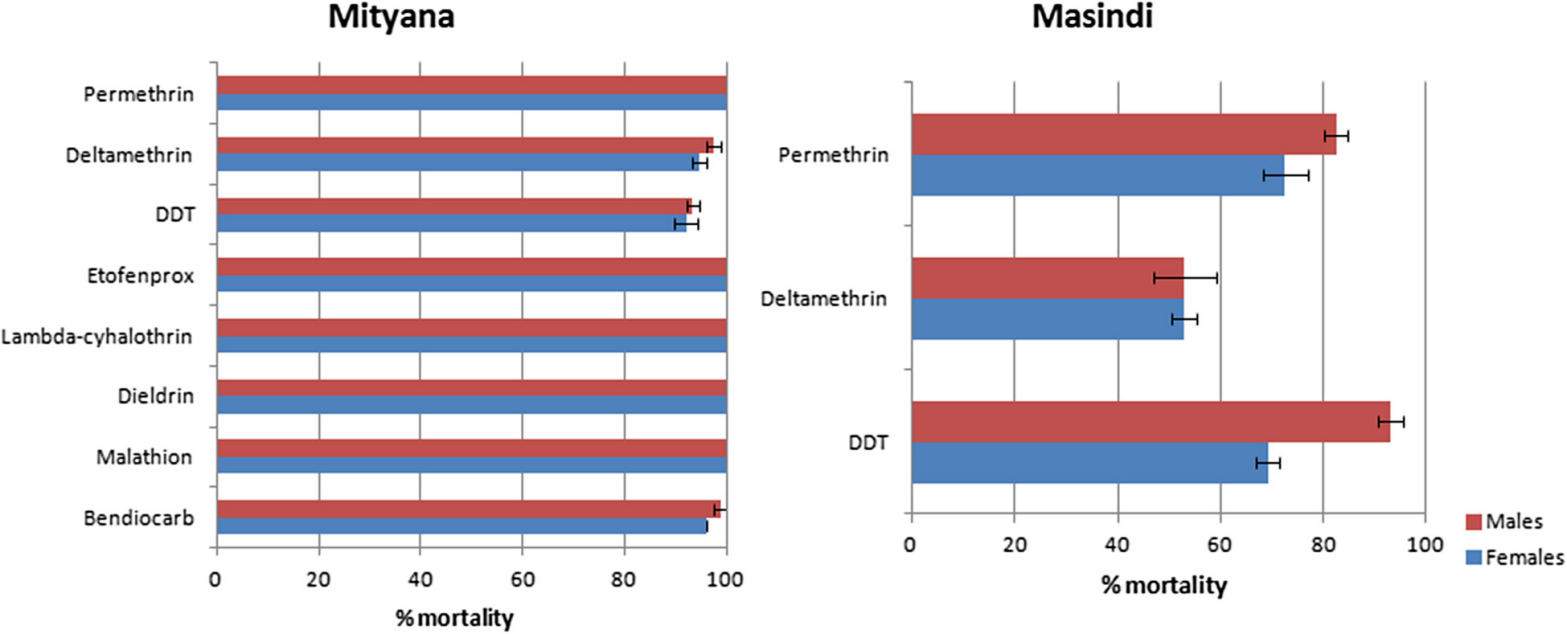
**Susceptibility levels in F**_**1 **_**adults from Mityana and Masindi after 1 hr exposure to insecticides.** The data shown are mean + SEM (n ≥ 4).

A total of 1753 F_1_ adults from all the three sites combined were exposed to various insecticides. *An. parensis* from Mityana exhibited a full susceptibility to most insecticides with 100% mortality observed for both males and females for the pyrethroids permethrin, lambda-cyhalothin and etofenprox. Similarly, a full susceptibility was observed for the organosphosphate malathion and the organochlorines dieldrin (Table 3; Figure 2). A reduced susceptibility was, however, observed for the pyrethroid deltamethrin (95 ± 1.3% mortality for females), for DDT (92 ± 2.3% mortality for females) and also for the carbamate bendiocarb (96 ± 0% mortality for females). A similar susceptibility profile was observed for the males (Table 3; Figure 2).

**Table 3:** Insecticide susceptibility levels by location after 1 hr exposure

**Insecticide**	**Sex**	**Mityana**		**Masindi**		**Ntungamo**	
		**n**	**% mortality**	**n**	**% mortality**	**n**	**% mortality**
Permethrin	F	75	100 ± 0	93	73 ± 4.3	20	100 ± 0
	M	75	100 ± 0	83	82 ± 2.1	22	100 ± 0
Deltamethrin	F	75	95 ± 1.3	75	53 ± 2.5	21	100 ± 0
	M	75	97 ± 1.3	70	72 ± 6.1	26	100 ± 0
DDT	F	75	92 ± 2.3	90	69 ± 2.3	15	63 ± 0
	M	75	93 ± 1.3	70	93 ± 2.5	17	100 ± 0
Lambda-cyhalothrin	F	75	100 ± 0	/	_	/	_
	M	75	100 ± 0	/	_	/	_
Etofenprox	F	75	100 ± 0	/	_	/	_
	M	75	100 ± 0	/	_	/	_
Bendiocarb	F	75	96 ± 0	/	_	/	_
	M	75	98.6 ± 08	/	_	/	_
Malathion	F	75	100 ± 0	/	_	/	_
	M	75	100 ± 0	/	_	/	_
Dieldrin	F	75	100 ± 0	/	_	/	_
	M	75	100 ± 0	/	_	/	_
Total	F	600		258		56	
	M	600		223		65	

/: not tested.

In Masindi where both *An. parensis* and *An. funestus* are present, resistance was observed to both type I (permethrin; 73 ± 4.3% mortality for females) and II (deltamethrin; 53 ± 2.5% mortality for females) pyrethroids and also to DDT (69 ± 2.3% mortality for females) (Table 3; Figure 2). Attempts were made to species identify survivors of insecticide exposure. Using PCR, all the 48 tested females were confirmed to be *An. funestus s.s..* This indicates that the *An. parensis* population in Masindi is similarly susceptible to these insecticides as the population from Mityana. Since there is a near ratio of 1:1 between the two species in Masindi, it can be deduced that the resistance level of *An. funestus* is probably twice the level recorded from these mortality rates (Table 3).

The F_1_ adults raised from Ntungamo were not enough (only 56 females + 65 males) for resistance profiling. However, mortality levels were not different from that observed in Mityana (Table 3).

**Figure 3:**
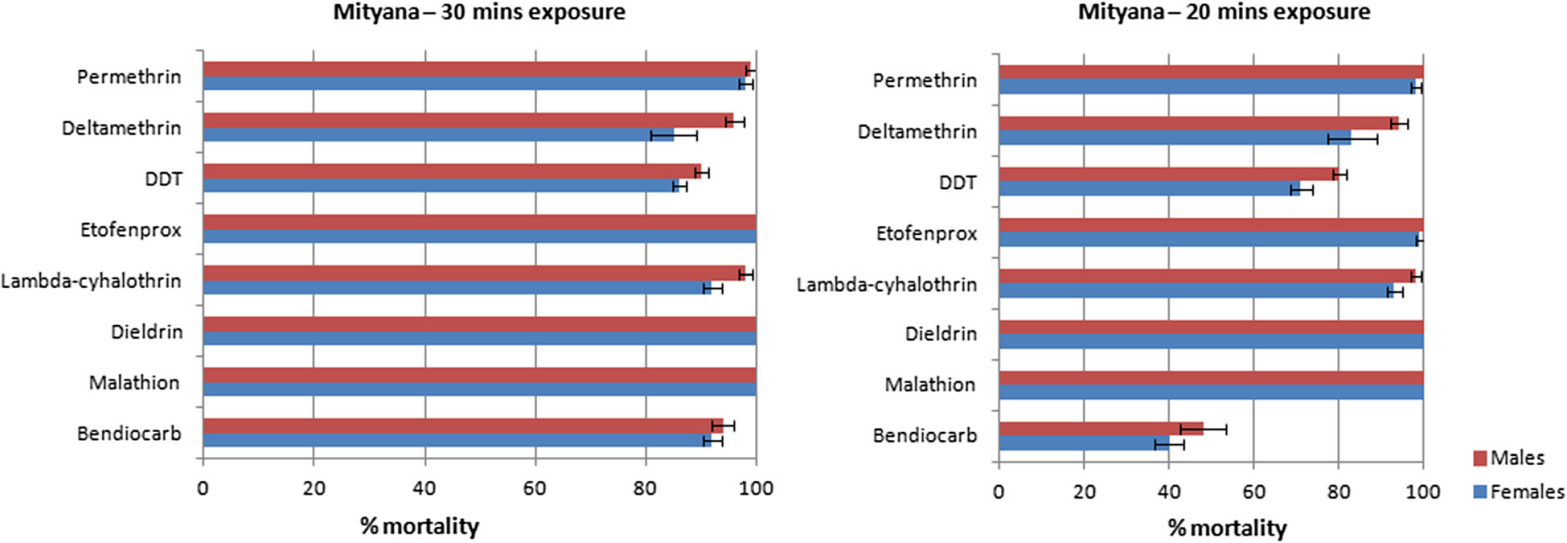
**Susceptibility levels of *An. parensis *from Mityana after 30 and 20 minutes exposure to insecticides.** The data shown are mean + SEM (n ≥ 4).

After observation of high mortality (>92% for both males and females) in the Mityana population and for all the insecticides, two pools of 1200 Mityana F_1_s each, were exposed for 30 and 20 minutes respectively to further assess the extent of susceptibility in this population. There was no remarkable difference in mortality for the 30 min exposure with all the insecticides (Figure 3). The trend drastically changed after 20 minutes exposure, particularly with bendiocarb for which > 50% of the population survived after 24 hours. Mortality was remarkably lower with deltamethrin and DDT after 20 minutes exposure compared to 1 hr and 30 minutes exposure (Figure 3).

### Trancription profiling of candidate resistance genes in *An. parensis*

**Figure 4:**
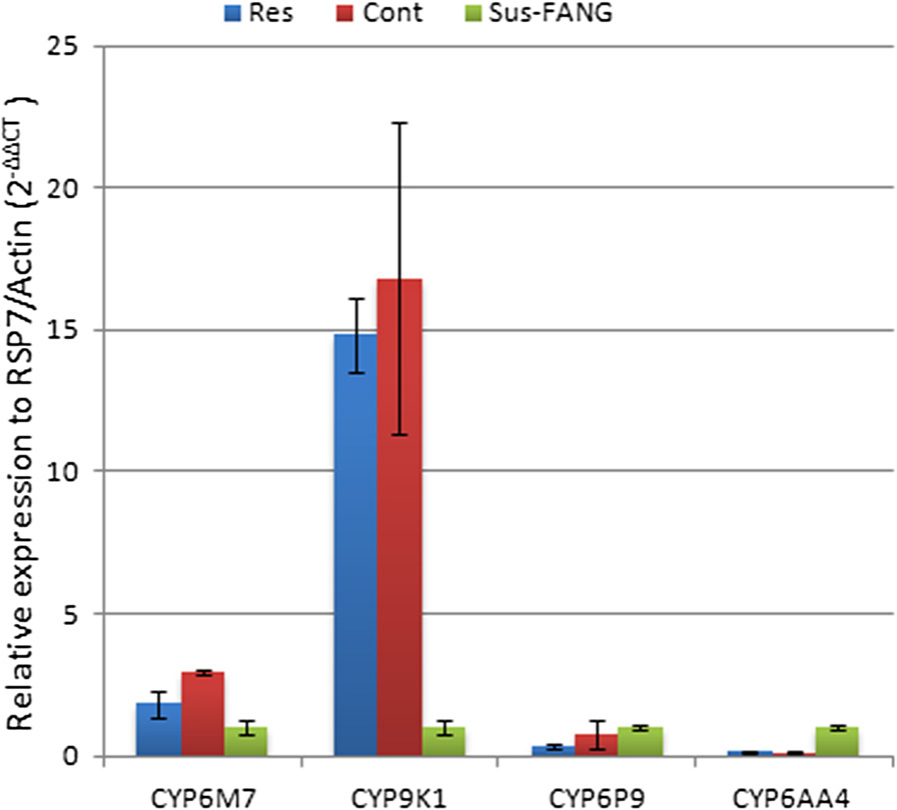
**Transcriptional profiling of candidate resistance genes by qRT-PCR in *An. parensis *from Mityana.** Mosquitoes alive after 30 minutes exposure to deltamethrin (Res) were compared to a control sample not exposed to insecticide (Cont) and to a susceptible laboratory strain (Sus-FANG) of *An. funestus*.

qRT-PCR analysis of the expression profiles in *An. parensis* of four candidate genes (CYP6M7, CYP9K1, CYP6P9 and CYP6AA4) previously associated with pyrethroid resistance in the sister species *An. funestus* s.s. [20,23] was successfully carried out with primers originally designed for *An. funestus*. It is unknown whether the CYP6P9 is duplicated in *An. parensis* as in *An. funestus*. Therefore, the primers used here for CYP6P9 were common to both duplicated CYP6P9a and CYP6P9b. Primers for all the four genes exhibited appropriate standard curve and amplification efficiency (between 90 and 110%), suggesting a high level of sequence conservation for these genes between the two species. None of the four genes was significantly over-expressed in the mosquitoes alive after 30 min exposure to deltamethrin (Resistant) than in those not exposed to insecticides (Control) (Figure 4). However, when the expression profiles of the four genes are compared with the fully susceptible FANG strain from *An. funestus s.s.,* (Sus-FANG), the cytochrome P450 CYP9K1 is around 15-fold over-expressed (P < 0.001) in both resistant and control *An. parensis* samples than in FANG. The major gene associated with pyrethroid resistance in *An. funestus s.s*., CYP6P9, is not significantly differentially expressed between the two species, while CYP6AA4 is rather significantly more expressed (7-fold; P < 0.01) in the susceptible FANG strain than in *An. parensis*.

## Discussion

Characterisation of members of the *An. funestus* group in Uganda has been very limited to date. In this study, we investigated the geographical distribution of members of this group, their contribution to malaria transmission and also their susceptibility to main public health insecticides.

### Species composition

In this study, of the nine to eleven species of the *An. funestus* group described to date, only *An.parensis* and *An. funestus s.*s were detected in Uganda. While the presence of *An. funestus s.s.* is well documented [2,11] this is only the second report of *An. parensis*. Indeed, *An. parensis* was previously identified in Western Uganda but only from outdoor collections [24]. Our findings show *An. parensis* to be abundantly present in Central (Mityana), South Western (Ntungamo) and North Western (Masindi) Uganda. *An. funestus s.s* is the main species of the group in the North and Eastern parts of Uganda from our collections and from previous studies carried out in these areas [2,11]. However, one cannot rule out the presence of other species of the group across the country because of the limited number of sites assessed. Additionally, the fact that this study only focused on indoor-resting mosquitoes could have prevented the detection of exophilic species of the group.

In this study, *An. parensis* was found abundantly resting indoors similar to previous reports in other countries such as Kenya [25] and South Africa [26], indicating that this species which was previously described as mainly exophilic [4,5,7], exhibits some plasticity in terms of its resting behaviour. The fact that *An. funestus s.s* and *An. parensis,* two species with such varying behaviour and vectorial capacity, as previously observed in Kenya [27], can be found indoors in sympatry is a serious concern to malaria vector control programs. *An. parensis* endophily as well as its sympatric occurrence with *An. funestus s.s,* such as in Masindi, is of significant interest since their varying behaviour [27] and infection rates could lead to misleading results on *An. funestus s.s.* if proper species identification is not carried out at the molecular level. This is the case in Masindi where the real level of resistance to insecticides in *An. funestus* has been underestimated because of the dilution from the sympatric susceptible *An. parensis* population. Additionally, even in areas where *An. parensis* is allopatric, its endophilic behaviour could lead control program managers to wrongly assume that *An. funestus s.s.* remains susceptible to insecticides. This may delay implementation of the much needed insecticide resistance management strategies to ensure the continued success of the insecticide-based control interventions. Mis-identification of vector species was already highlighted as one of the potential problems that led to vector control failure in the Garki project [28]. Our study further highlights how essential it is for every control program to ensure that molecular techniques together with morphological keys are used for reliable species identification during their surveillance activities.

### Sporozoite infection rate

Previous reports [25,29,30] have not incriminated *An. parensis* in malaria transmission, however, given that a high number of indoor blood fed female *An. parensis* were collected, there was a need to establish whether it plays a role beside *An. funestus* in malaria transmission. This study confirms previous reports with the detection of sporozoite positive samples only in *An. funestus s.s* and not in *An. parensis.* The positive cases observed for *An. parensis* by TaqMan are probably due to false positives as also observed for this species in South Africa when using ELISA-CSP [26]. However, further investigations involving large scale screening of *An. parensis* for *Plasmodia* is highly recommended before this species is considered a non-malaria vector. The infection rate of 5.3% observed in *An. funestus s.s.* is similar to levels commonly reported for this species across Africa [13]. Although the source of blood meal was not tested in this study, the fact that cattle and other domestic animals were very few in Mityana, suggests that humans could have been the prime source of the blood meal for the collected females.

### Insecticide resistance

In Mityana, the susceptibility of the *An. parensis* population to most insecticides is further supported by the low LT50 of this population for all insecticides, (<20 min, except for the carbamate bendiocarb). *An. parensis* susceptibility to insecticides in Uganda is similar to what was observed for this species in the KwaZulu Natal region of South Africa, where out of four tested insecticides, *An. parensis* was fully susceptible to three, specifically permethrin, DDT and bendiocarb [26]. However, a suspected resistance was observed against deltamethrin in the *An. parensis* population of KwaZulu Natal similar to the moderate resistance also observed for the same insecticide in Mityana. Overall, the similarity of susceptibility patterns between two populations of this species from East and Southern Africa suggests that *An. parensis* is most likely still susceptible to most public health insecticides across its range of distribution in Africa. The susceptibility in *An. parensis* contrasts with the high resistance levels observed in *An. funestus* in this study and other populations across Africa [14,31-34]. A similar contrast in insecticide resistance profiles was reported recently between *An. gambiae s.s* and *An. arabiensis* in Cameroon [35]. Such differences in susceptibility patterns suggest that *An. parensis* is only partially endophilic and under less selective pressure from public health control interventions than the predominantly endophilic *An. funestus*. Indeed, *An. parensis* has been found in the past to be mainly exophilic and exophagic [27] and therefore under less selection pressure than *An. funestus*. Since both species have similar breeding sites, it is likely that resistance may have been more selected in *An. funestus* due to its indoor resting behaviour, leading to exposure to public health insecticides, which may not have been the case for the more exophilic *An. parensis* until recently. However, the endophilic behaviour displayed by *An. parensis* in this study is likely to increase the selection pressure on this species particularly with the recent scaling up of indoor residual spraying in Uganda. This situation is likely to lead to the rise of resistance in *An. parensis* in the future. Therefore, the susceptibility profile of this species should be monitored.

The low fold-change observed for four candidate resistance genes between the *An. parensis* samples that survived 30 min exposure to deltamethrin and the control non-exposed mosquitoes, further supports the overall susceptibility observed in this species contrary to *An. funestus*. The 15-fold over-expression of the cytochrome P450 gene, CYP9K1, in the *An. parensis* samples compared to the susceptible FANG strain could suggest that this gene may play a role in the observed deltamethrin resistance. However, because this difference could also be due by interspecies variations in gene expression, further work is needed to validate CYP9K1 involvement. It cannot be ruled out also that the genes responsible for the reduced susceptibility to deltamethrin in *An. parensis* could be different to that acting in *An. funestus* as observed between *An. arabiensis* and *An. gambiae*[36]. Indeed, it was recently shown that the metabolic resistance observed in a field population of *An. arabiensis* in Chad is probably conferred by the CYP6P4 cytochrome P450 gene and not the CYP6P3 or CYP6M2 genes which are the major resistance genes in the sister species *An. gambiae*[36]. Therefore, other methods, notably genome-wide gene expression profiling, could be used to detect these *An. parensis* specific resistance genes.

## Conclusion

The significant difference in the resistance profile and potential role of malaria transmission of *An. parensis* and *An. funestus s.s* reported in this study is a reminder of the importance of accurate species identification of malaria vectors in any vector control program. Such molecular identification should be part of any monitoring and surveillance activities in order to ensure that the impact of control interventions on vector populations is adequately assessed.

## Competing interests

The authors declare that they have no competing interests.

## Authors’ contributions

CSW conceived and designed the research. CM and JB carried out the sample collection; CM and JMR performed the WHO bioassays; CM and HI performed the molecular analyses; JB and LGM contributed toward data analysis and significant insights; CM and CSW drafted the manuscript with contribution from all the authors. All authors read and approved the final manuscript.
